# Precision Monitoring of Honey Bee (Hymenoptera: Apidae) Activity and Pollen Diversity during Pollination to Evaluate Colony Health

**DOI:** 10.3390/insects14010095

**Published:** 2023-01-16

**Authors:** Aimee C. McKinnon, Luke Collins, Jennifer L. Wood, Nick Murphy, Ashley E. Franks, Martin J. Steinbauer

**Affiliations:** 1 Department of Ecology, Environment and Evolution, La Trobe University, Melbourne, VIC 3086, Australia; 2 Department of Physiology, Anatomy and Microbiology, La Trobe University, Melbourne, VIC 3086, Australia; 3 Research Centre for Future Landscapes, La Trobe University, Melbourne, VIC 3086, Australia

**Keywords:** pollination, pesticides, metabarcoding, nutrition

## Abstract

**Simple Summary:**

Honey bee colonies deployed for pollination must be healthy to meet industry demand. Monitoring the activity of bees can indicate hive status but it is a complex task because bee colonies are inherently variable and require a holistic approach. In this study, we endeavored to monitor honey bee hives in the field using a combination of remote surveillance to quantify flights and by assessing the risk of pesticide exposure to bees from the pollen they collect. In Australia, vast almond orchards are grown as monoculture and in a semi-arid region, and so a lack of floral diversity may also impact bee health during pollination. Almonds are not produced without honey bees, and so it is essential to protect bee colonies, to ensure that there will be sufficient hives available in the following years. We showed that honey bee activity can be measured in the field to test for differences between hives. No insecticides were detected in pollen but combinations of fungicide residues were, although the individual concentrations were likely not hazardous. Floral diversity was perhaps less important for promoting bee activity compared to pollen availability, however, flowering weeds and trees in hedgerows may be important to help support hives.

**Abstract:**

Certain crops depend upon pollination services for fruit set, and, of these, almonds are of high value for Australia. Stressors, such as diseases, parasites, pesticides, and nutrition, can contribute to honey bee *Apis mellifera* L. colony decline, thereby reducing bee activity and pollination efficiency. In Australia, field studies are required to monitor honey bee health and to ascertain whether factors associated with colony decline are impacting hives. We monitored honey bee colonies during and after pollination services of almond. Video surveillance technology was used to quantify bee activity, and bee-collected pollen was periodically tested for pesticide residues. Plant species diversity was also assessed using DNA metabarcoding of the pollen. Results showed that bee activity increased in almond but not in bushland. Residues detected included four fungicides, although the quantities were of low risk of oral toxicity to bees. Floral diversity was lower in the pollen collected by bees from almonds compared to bushland. However, diversity was higher at the onset and conclusion of the almond bloom, suggesting that bees foraged more widely when availability was low. Our findings suggest that commercial almond orchards may sustain healthier bee colonies compared to bushland in early spring, although the magnitude of the benefit is likely landscape-dependent.

## 1. Introduction

Bees are important pollinators of plants in both natural and agricultural ecosystems. An estimated 65% of crops worldwide benefit directly or indirectly from animal pollination; however, honey bees are predominantly used for pollination services because of their availability, generalist floral preferences, and ease of management [1,2]. Honey bees are especially important for broad-acre crop and orchard species. In the 2019–2020 season, Australia exported 76,556 tonnes of almond kernels valued at AUD 772.6 million, demonstrating significant market growth as 60,894 tonnes of almonds were exported in 2018–2019 (Almond Board of Australia. 2019/2020 Almond Insights). The recent expansion of almond orchards has increased the total area to over 53,000 hectares, valued at more than AUD 1 billion. The highest economic value of managed pollination services occurs in the state of Victoria, and there is increasing demand for pollination services in Victoria because of the size and location of the almond industry, with a requirement of between 150,000–200,000 hives during bloom. Victoria is also host to other pollination-dependent industries, such as pome fruits and summer fruits, contributing further to the demand. The expansion of pollination-dependent industries requires more hives than Victoria has access to; therefore, there is significant seasonal movement of managed bee hives from other regions to meet the demand. Consequently, there is a need for more studies that identify the risk factors associated with colony health in Australian agroecosystems to continue to support the requirement for healthy honey bee colonies year after year [3,4].

Monitoring the hive activity and detection of transmissible diseases is important to maintaining pollination efficiency [5]. Precision apiculture is a concept involving the real-time monitoring of beehives and is generally intended to help apiarists rapidly identify different colony states or unusual behaviour, such as swarming, brood decline, colony collapse from pathogens, queenlessness, or starvation [6]. To accomplish this, data are collected at different scales. In the apiary, meteorological parameters, such as temperature, relative humidity, light, wind speed, and rainfall can be acquired in real time. Video recording may be used to aid in the detection of apiary disturbance via audio or motion detectors. Additionally, the weight of the hive, the number of individual bees entering and exiting the colony, or the number of bees in flight around the hive entrance can help inform the status of colonies [7]. Novel technologies have been recently developed to measure specific parameters, such as colony activity, weight, acoustics, and internal temperature, thereby indicating health [6].

Whereas various interacting biological stressors, such as diseases, parasites, nutrition, and cold stress, may contribute to the rapid decline in colony health [8,9], there is also evidence that pesticides applied to crops can have a negative impact on managed bees [3,10,11,12,13,14]. Several studies have reported sublethal effects to honey bees from pesticide exposure, although these have predominantly been conducted within controlled settings [4] and have frequently involved the direct poisoning of bees with sublethal concentrations of specific pesticides [15]. Large-scale studies have been conducted in the UK and Europe to quantify pesticide residue levels from pollen collected by honey bees directly [16,17,18]. In North America, insecticide residues, forager activity, and colony health have also been monitored in the field [3,19,20]. In Australia, there are studies that have quantified pesticide residues from bee-collected pollen-servicing canola crops [21,22], but there are no studies that have also monitored activity or health during potential exposure events. The absence of the major mite parasite *Varroa destructor* Anderson and Trueman (Mesostigmata: Varroidae) in Australia also presents an opportunity to monitor the effects of chronic pesticide exposure without the potentially confounding effects of significant mite stress and miticide treatment [23].

To confound the threat further, colony activity and health are also known to be strongly associated with nutrition [24,25] and climate variables [26]. Access to excellent nutrient resources may also mitigate some of the sublethal effects of pesticide exposure [27]. Foraging bees ingest nectar and collect pollen, eventually storing the latter with a mix of regurgitated nectar, saliva, and honey to seal within the comb. Eventually, nurse bees in the colony provide this stored resource, known as bee bread, to develop brood. Large scale monoculture has been suggested as a potential stressor for honey bee colony health [25,28], particularly where pollen quality is poor or insufficient, as brood development and parasite tolerance relies on nutrients derived from either (1) high quality pollen, or (2) access to a diversity of floral resources [20,29]. For this reason, DNA metabarcoding is a method that has been used to investigate foraging by bees, including in Australia, through the identification of plant taxa present in pollen [30,31,32,33,34]. Pollen DNA metabarcoding is a high-throughput method that can detect significantly higher quantities of taxa compared to conventional microscopy and with greater confidence [32,35,36], providing the means to evaluate the floral preferences or visits of pollinators with improved accuracy.

In Australia, field studies are required to investigate what factors may influence domestic honey bee colony activity and health, and, in turn, how these may impact pollination efficiency. Although *V. destructor* is still absent in Australia, incursion events continue to threaten the industry, and, moreover, honey bee pathogen surveillance is limited (but see Brettell et al. [5]). The aim of our study was to assess for potential impacts to honey bee colony health during pollination services. We used non-intrusive commercially available bee colony video surveillance (Eyesonhives, Keltronix Inc., Santa Barbara, CA, USA) to quantify forager and emerging bee activity in the field, both during and after the pollination services of almond trees in northern Victoria, Australia. According to the Eyesonhives developers, daily activity peaks recorded by the technology at the hive entrance may be associated with emerging juvenile honey bees making their initial orientation flights, based on video footage. This ‘orientation signal’ in the data is likely indicative of bee health, since healthy colonies continue to produce brood, whereas diseased/dying colonies tend to decline in brood production prior to collapsing. Whether the decline in brood activity can be captured is dependent on how rapidly this occurs and the severity of the cause; however, this data may be very useful in studying sublethal effects to honey bee colonies in the field. Bee-collected pollen was periodically sampled from colonies to test for the presence of pesticide residues. Furthermore, we analyzed plant species diversity in the bee-collected pollen as a potential indicator of nutrient quality, using DNA metabarcoding of the ITS2 marker to investigate the suitability of monocultural floral resources (almond) for colony health. By combining these different approaches, we sought to provide the first longitudinal and quantitative data on the cost to hives of the commercial honey bees of the pollination of almonds in Australia. Since commercial almond orchards in Australia have been established in climatic regions naturally dominated by Mallee vegetation, we placed honey bees in this habitat as our control. Mallee is a unique Australian habitat, the name of which is derived from the growth form of the eucalypts endemic to this large region [37]. We also monitored hive health when honey bees were returned to the Red Gum dry sclerophyll forest near Melbourne for the summer. 

## 2. Materials and Methods

### 2.1. Remote Activity Monitoring

**Figure 1:**
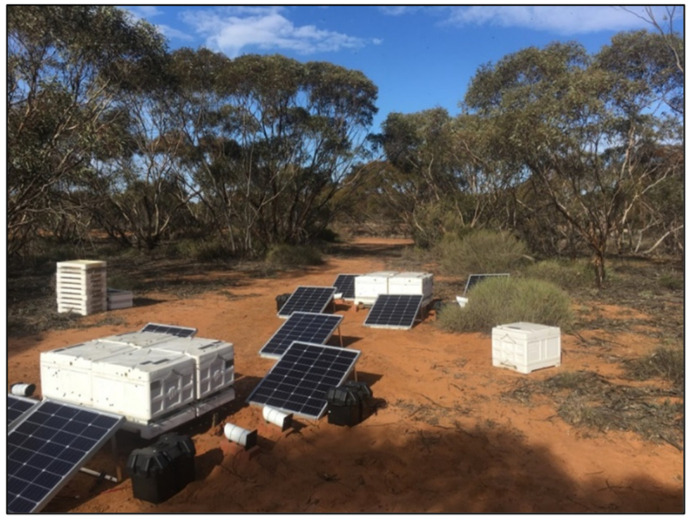
Image of honey bee hives with bottom-mounted pollen traps installed and the Eyesonhives cameras set up with solar power supply for the remote recording of the number of bee flights at the hive entrance.

Honey bee flight activity was quantified at the hive entrance using Eyesonhives remote surveillance technology developed by Keltronix Inc (Santa Barbara, CA, USA). Briefly, Eyesonhives traces the flights of bees and counts them using a specifically designed algorithm. Counts are calculated as average bees per second (BPS) every 30 s and uploaded to the Eyesonhives cloud-based server via a Wi-Fi connection. The data uploaded included periodic video content that captures 30 s long footage corresponding to the algorithm-detected differential activity fluctuations. Additionally, local climate data (temperature, humidity, weather conditions, wind speed, wind direction) were automatically acquired from the nearest Australian Government—Bureau of Meteorology weather station (Swan Hill, ID: 94843). For all experiments, the cameras were secured to face hives at 40 cm from the entrances and connected by USB to individual 24 v 26 amp hour batteries inside weatherproof housings. Each battery was kept fully charged by an 80 v solar panel (Figure 1). Portable USB modem units were installed in selected battery housings and were able to serve up to five cameras per site depending on network availability.

### 2.2. Bee Count Data Validation

Pilot studies were conducted to test whether the Eyesonhives BPS count data could adequately quantify true differences between hives in a field experiment. 

Initially, the validation of count data was evaluated by performing tally counts of honey bee flights in the Eyesonhives video footage. Three Eyesonhives remote camera kits were affixed to monitor three beehives at La Trobe University campus (Victoria, Australia), with each colony containing approximately 5 frames of bees. Tally counts were subsequently made from 14 randomly selected videos uploaded between January–February 2019. Honey bees were only counted when they fully exited or returned to the hive entrance in flight to simulate the Eyesonhives flight counts. To obtain the BPS count by tally, the total number of honey bees flights counted per video was divided by the 30 s video interval. Each video tally count total was replicated three times per video to provide a mean count. The relationship between the tally count data and the Eyesonhives count data was analyzed using a base linear model in R version 3.5.1.

#### Assessing Activity between Strong and Weak Colonies

A preliminary experiment was conducted in autumn (15 April 2019–12 May 2019) to ascertain the sensitivity of the Eyesonhives cameras for detecting differential activity between hives of different colony size. A total of 14 cameras were placed to monitor hives located at two sites in Wonga Park (−37.75881, 145.26137) and Warranwood (−37.76601, 145.25519), Victoria, that had active honey bee colonies ranging in size or ‘strength’. Strong colonies were designated as hives containing 12–13 full frames of bees (FOB), and weak colonies had 5–6 FOB. Each site had seven hives consisting of three strong colonies and four weak colonies arranged randomly but adjacent to each other. Additionally, all hives were given sucrose syrup (1:1) once per week for 4 weeks during the trial, which was delivered in feeding frames that are internal to each hive. For each site, 4 hives were given syrup mixed with the chlorine treatment [2 g/10 L] dichloroisocyanuric acid, which is routinely used to treat bee-drinking water provided during spring pollination. 

Analysis focused on the effect of hive strength on hive activity. BPS count data were converted to the mean number of honey bee flights counted by the Eyesonhives algorithm, per 30 s intervals, summed every hour from 8 am–6 pm daily. The preliminary investigation of the hive activity data detected a strong non-linear diurnal trend in hourly bee counts. To standardize daily comparisons of bee counts, we decided to focus on the time of day when bee activity was highest. We used generalized additive mixed models (GAMMs) in R (v. 3.5.1) with package mgcv (v. 1.8-31; Wood 2011) to examine the effect of time of day on bee counts while accounting for repeated measures within hives. Generalized additive models are a form of generalized linear model in which the relationship between the response variable, and at least one predictor is represented by an unknown smooth function, thus facilitating non-linear relationships between response and predictor variables. GAMMs allow for the inclusion of random effects into the generalized additive model framework. We fitted time of day as a smooth term to account for the non-linear diurnal trends in bee counts and hive ID as a random intercept to account for repeated sampling from the same hives. Model predictions were then plotted to identify the temporal peak in bee activity. A GAMM was used to test the effect of hive strength on bee counts for the time of day when bee activity was highest, based on the prior modelling, while accounting for environmental covariates. Fixed effects included the hive strength and the sugar syrup treatment. Site temperature (°C) and wind speed (Km per hour) were included as smoothing terms to account for deviance in activity, resulting from variation in weather conditions between sites and hives. We fitted unique smoothing terms for the different levels of hive strength as relationships between bee counts and weather appeared to vary between weak and strong hives. Relative humidity data correlated with temperature and, as such, was excluded from the analysis. Other factors that were considered and investigated independently for effects to forager activity levels were queen age and the number of honey frames in each colony (results not reported).

### 2.3. Spring Pollination Trial and Summer Recovery

For the spring pollination trial, ten honey bee hives with active, eight frame colonies were arranged in two blocks, consisting of five hives facing east and five facing west, arranged at random on a border of two adjacent (situated to the east and west) almond orchards at Lake Powell, Victoria (−34.73878, 142.92039) from 27 July to 19 September 2019. A further 10 hives were arranged in east/west facing blocks in Mallee bushland at Hattah, Victoria (−34.75943, 142.28542). Hattah encompasses both Lowan Mallee and Murray Mallee (https://vicflora.rbg.vic.gov.au/static/bioregions/ accessed on 25 August 2022). All hives were rigged with Eyesonhives cameras and were remotely monitored for the duration of the trial. Hive placement was constrained by the need for network coverage to access environmental data and upload counts remotely. Consequently, hives were arranged at two sites with hives grouped by blocks facing west/east at each site.

To further evaluate honey bee activity and health after pollination, all surviving bee colonies were relocated after 19 September 2019 to Red Gum dry sclerophyll forest habitat at Warranwood (−37.76601, 145.25519), Victoria at the conclusion of spring pollination and monitored continuously with the Eyesonhives cameras until 5 January 2020. 

### 2.4. Activity Data Analysis for Spring and Summer

For the spring dataset, the aim was to compare bee activity between the sites, however, we were interested in analyzing whether differences in the pollen collected by bees (e.g., due to residues, and/or floral species composition) influenced activity during the pollination period. The activity data were, therefore, partitioned for the intervals prior to pollen sampling days, so that hive activity during these intervals could be used as a proxy for pollen collection activity. The BPS count data were converted to the sum of honey bee flights counted by the Eyesonhives algorithm per 30 s intervals. The means of the counts were then calculated for each hive for the four days prior to every corresponding pollen sampling event of 16, 23, 32, and 37 days after placement of the hives at their respective site (see pollen sampling method below). 

Initially, the mean counts per 30 s intervals were graphed over pollen sampling intervals for each site and block to assess activity variability over time using ggplot2 (v. 3.3.0). Hive activity was then modelled for statistical differences over days and by site, using a generalized linear mixed model (GLMM), using the glmer function in the package lme4 (v. 1.1-21) in R (v. 3.5.1). The GLMM was formulated on the Poisson error distribution for count data, using a log link function and fit by maximum likelihood (Laplace approximation) for the mean bee counts per sampling interval (these were rounded to integers). Fixed effects were categorical and included the pollen sampling interval (days after hive placement), site (almond, bushland), and the block (east, west). To incorporate the repeated measures dependency among the observations of the same colony, we used hive as a random intercept. Model assumptions were checked by plotting residuals versus fitted values, and by checking for overdispersion.

For the summer activity dataset, honey bee activity was assessed using GAMMs after converting the BPS count data to the mean number of honey bee flights, as described previously for the autumn pilot study. The bee count data were restricted to data recorded at windspeeds below 25 Km/h to reduce excess counts recorded from wind motion/shadows. Hives 8, 9, and 12 had high activity and were omitted when estimating daily general activity and the activity peak. Activity was summed by the hour, daily, for the month of December (2019) and differences in activity between hives generally and between hives from former sites were then compared. The summer activity data were graphed, based on an estimated daily temporal honey bee activity predicted in a GAMM for all hives and also for each site. Fixed effects included the former site (almond/bushland) and the block (east, west). Smoothing terms included temperature (°C) and wind speed (Km/h), with unique smoothing terms for the different levels of former site (almond/bushland). Model assumptions were checked by plotting residuals. 

### 2.5. Pollen Collection

Pollen samples were obtained from the study hives between 27 July and 19 September 2019, at 16, 23, 32, and 37 days after placement in either the almond orchard (Lake Powell, Victoria) or in bushland (Hattah, Victoria, Australia). Hives were fitted with complete bottom-mounted pollen traps (Nuplas Apiary Supplies, Swan Hill, Australia) to collect corbicular pollen. The wire mesh size of 5 mm only partially stripped pollen from returning foragers to allow brood in the hives to be fed until the final sample was taken at 37 days. Pollen trap draws were emptied at each sampling time. 

### 2.6. Pesticide Residue Analysis

#### 2.6.1. Pesticide Detection from Pollen

For the residue analysis, samples were pooled into four 50 g samples at each time point (16, 23, 32, 37 days from 27 July 2019) for each block of five hives: groups A and D were in the bush and groups B and C in almond. Comprehensive pesticide residue analyses were conducted by Agrifood Technology (Werribee, Victoria, Australia) using a standardized multi-residue QuEChERS (quick, easy, cheap, effective, rugged, and safe) method for extracting and detecting pesticides following liquid chromatography/mass spectrometric analysis [38]. The Agrifood Technology laboratory evaluated pollen for approximately 150 different substances currently used in agricultural practices in the state of Victoria, Australia.

#### 2.6.2. Residue Evaluation

To estimate the potential hazard to bees from contaminated pollen loads, the pollen hazard quotient (PHQ) was calculated following a method described by Stoner and Eitzer [39]. Briefly, this method provides a simple way to calculate risk based on known LD_50_ values for honey bees that are readily available online. The concentrations of each residue found in a sample (μg/kg) were first divided by the LD_50_ (honey bee oral; μg/bee) found for each respective substance. The LD_50_ values were obtained from the University of Hertfordshire’s pesticides properties database (http://sitem.herts.ac.uk/aeru/ppdb accessed on 21 November 2019). Total PHQ per sample was calculated as the sum of all PHQs of the pesticides in the pollen sample for a given time point. The PHQ sum, therefore, does not consider the risk of synergistic reactions that may occur between the residues when combined. To evaluate for sublethal effects, a PHQ value must exceed a threshold of 50 to be considered of concern. This threshold is based on the estimated daily pollen consumption rate of 9.5 mg by a nurse bee [39,40,41], whereby a PHQ of 50 corresponds to 0.05% of the LD_50_ consumed per day, resulting in 0.5% of the LD50 in an average 10-day nursing period [16,39].

### 2.7. Metabarcoding

#### 2.7.1. DNA Extraction and Sequencing

The DNA marker gene, ITS2, was sequenced with 15% phiX control on the Illumina MiSeq platform using paired end sequencing (2 × 300 bp) to determine plant species diversity in forager bee-collected pollen. From each hive, 5 mL volume samples of corbicular pollen pellets were collected at each sampling time from the top 5 cm of the heap in the trap and individually mixed using a sterile mortar and pestle in 15 mL deionized H_2_O, to acquire a homogenized pollen suspension. From each sample suspension, 1 mL was taken and stored at −20 °C overnight, providing a total of 80 pollen suspensions. Prior to DNA extraction, the suspensions were thawed at ambient temperature and then centrifuged at 10,000× *g*, the supernatant discarded, and the pollen air-dried for four hours in a laminar flow cabinet. To extract genomic DNA from the pollen, 50 mg of each sample was resuspended in the tissue disruption tubes of the DNeasy Plant Pro kit (Qiagen, Melbourne, Australia) with lysis buffer. The pollen was then pulverized using a custom vortex bead-beater adapter kit for 15 min at full speed. Pollen DNA was then isolated using the DNeasy Plant Pro Kit, according to the instructions provided. Genomic DNA was also extracted by the same method from the following samples to serve as experimental controls for sequencing: (1) a sample of pollen supplement, (2) an almond flower sampled from the trial site, (3) elution buffer, and (4) a honey bee abdomen. All DNA samples were normalized to working concentrations of 5 ng/µL and stored at −20 °C until required. 

The target amplicons were prepared according to the Illumina protocol by PCR for sequencing using 12 nt unique indexing barcodes and established plant primer sets, which included locus specific Illumina overhang adapter sequences (Illumina 16S Metagenomic Sequencing Library Preparation guide #15044223). Primers targeted ITS2, which provided a 300–460 bp amplicon using ITS2-S2F forward 5′-ATGCGATACTTGGTGTGAAT 3′ and ITS4 reverse 5′-TCCTCCGCTTATTGATATGC 3′ primers [42,43]. PCR amplifications were performed in 25 µL reactions using a CFX Connect Real-Time PCR Detection System (Bio-Rad, Sydney, Australia). Reaction mixtures contained: KAPA HiFi HotStart DNA Polymerase (0.5 U per 25 µL reaction) in a proprietary reaction buffer (KAPA HiFi HotStart ReadyMix, Roche, Sydney, Australia), which contains dNTPs (0.3 mM of each dNTP at 1X), MgCl_2_ (2.5 mM at 1X), and stabilizers; 0.5 pM of each primer; and 10 ng of gDNA sample. Thermocycler parameters for the amplification of the ITS2 region were set as follows: initial denaturation at 94 °C for 5 m, 30 cycles of 98 °C denaturation for 10 s, annealing at 50 °C for 30 s, extension at 72 °C for 45 s, and a final extension step at 72 °C for 10 min. Sequencing libraries were pooled equimolarly based on PCR amplification. Libraries were quantified on a Qubit (Thermo Fisher Scientific, Melbourne, Australia) and diluted to 2 nM.

#### 2.7.2. Data Processing and Taxonomic Assignment

After sequencing, the raw sequence reads were sorted by indices and trimmed for quality using the UPARSE clustering pipeline (USEARCH version 11; https://drive5.com/usearch/manual/uparse_pipeline.html accessed 12 January 2020). Reads were quality filtered by discarding reads with total expected errors >1.0. Delineation of taxa was achieved by first denoising reads into amplicon sequence variants (ASVs) via the “unoise3” command [44]. ASVs were then sorted by sample into a count table. 

All ITS2 ASV sequences were queried against the NCBI nucleotide database. The top three basic local alignment search tool (BLAST) hits that had >98% match with an e-value of 1 × 10^−5^ were selected and compared against the Atlas of Living Australia plant database (https://www.ala.org accessed on 15 February 2020) and the Royal Botanic Gardens Victoria VICFLORA database (https://vicflora.rbg.vic.gov.au accessed on 15 February 2020). ASVs assigned to the same taxa (species) were merged to create an operational taxonomic unit (OTU) table for downstream analyses, while ASVs that were not assigned to species level, and instead to a higher taxonomic rank (i.e., genus, family, order), were labelled instead with the corresponding Genbank accession number that they matched. Following this method, ASVs with a genetic similarity <96% were manually assessed using a neighbour-joining tree in MEGAN 6 Community Edition [45] and then also assigned into a single OTU when their divergence was <5% [46]. Individually querying each hit in this way provided the identification of plant genera and sometimes species that are reported to be present in the Sunraysia/Mallee region of Victoria, which enabled the assignment of taxa to each unique ASV set of consensus sequences accordingly. ASVs with no hits were omitted from subsequent biodiversity analyses, as were a few ASVs not assigned to plant taxa, such as three fungal genera hits. Experimental control samples were contrasted against all biological samples to ascertain spurious sequence reads and to confirm ASV taxonomic assignment, for example, for almond (*Prunus dulcis*), which were highly represented in reads. 

### 2.8. Statistical Analysis of Species Diversity

Statistical analyses of biological sample data were conducted in R Bioconductor (version 3.12) using packages’ phyloseq v1.34.0 [47] and microbiome v1.12.0 [48]. The samples were rarefied to a minimum read depth of 4300, which removed additional spurious ASVs from the dataset. Alpha diversity indices Chao1, Shannon’s H, and Simpson’s evenness were calculated and compared separately for each site (almond orchard versus bushland) and for each sampling interval. A Shapiro–Wilk normality test was conducted on the various alpha diversity indices to test for normality, and a Bartlett test determined that variances were not homogenous. Because data were not normally distributed, a Kruskal–Wallis rank sum test was used to test for significance in Chao1, Shannon, and Simpsons diversity indices for species within (1) sites and (2) for days after placement. 

Beta diversity analyses were then conducted to compare plant species compositions, initially by using nMDS with the Bray–Curtis distance method for normalization, to investigate the possible dissimilarities of plant species communities by site of hives and days after placement. Additionally, nMDS were conducted on subset data to visualize species communities by block and days within sites. PERMANOVA was performed using the adonis function in R to test for significant differences between plant species communities for pollen samples grouped by site, block (east versus west), days after placement, and number of bees (summed flights for each pollen collection interval by hive based on Eyesonhives data). ASVs were then merged by taxonomic rank (genus and/or family) to evaluate the composition of plant families and genera between sites and sampling intervals by relative abundance, using the tax_glom function in the package microbiome (v1.12.0). 

## 3. Results

### 3.1. Bee Count Data Validation

**Figure 2:**
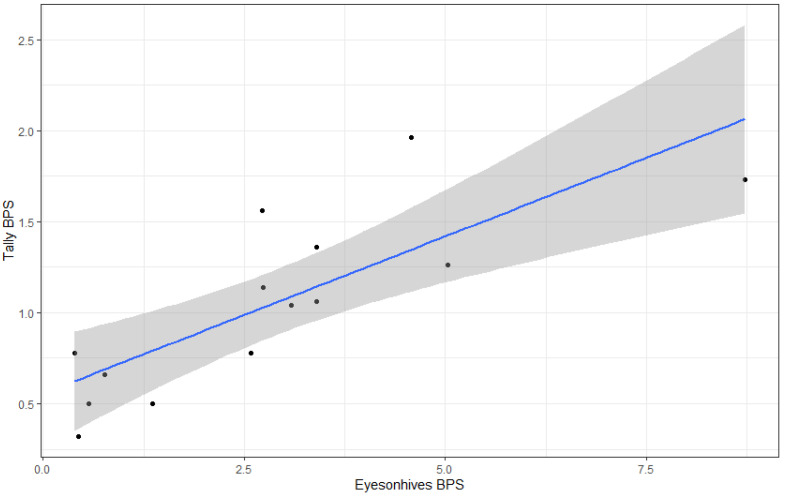
Linear regression for average bees per second (BPS) with 95% confidence interval indicated in grey represented with a tally counter (Y axis) compared against average BPS recorded by Eyesonhives cameras (X axis) (tally BPS = 0.56 + 0.17 × Eyesonhives BPS).

The first pilot study conducted to test the accuracy of the Eyesonhives bees per second (BPS) counts (analyzed as average sum per 30 s intervals) showed a significant positive relationship with tally counts (per 30 s intervals), with an adjusted R^2^ = 0.603; F (_1_, _12_) = 20.74, *p* < 0.001. (Figure 2). The slope of the fit between the Eyesonhives counts and tally counts was 0.17, indicating that the automated counts were ~5× greater than the actual counts per 30 s interval. 

**Figure 3:**
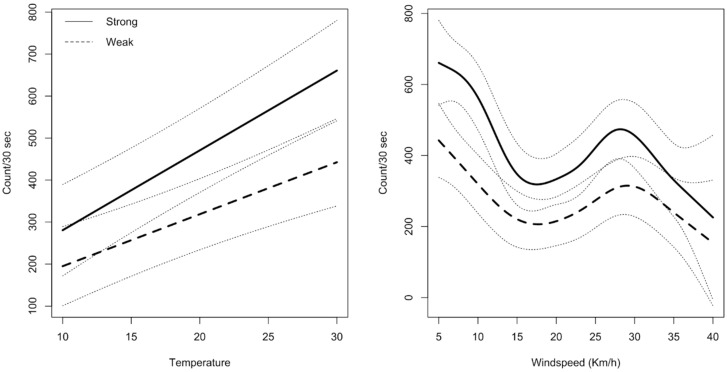
Estimated mean honey bee counts per 30 s with 95% confidence intervals, based on counts at 1400 h analyzed against temperature (°C) and windspeed (Kmph) for strong hives (solid line) versus weak hives (dotted line) in autumn.

The modelling of daily hive activity in autumn (based on the fitted GAMM) predicted that bee activity was generally highest between 1200 and 1400 h, but most consistent across all hives at 1400 h. Therefore, using the mean bee counts/30 s at 1400 h, a significant difference in activity between strong and weak hives (*p* = 0.002) was detected (Table 1). This difference was noticeably greater with rising temperature (°C) and windspeed (Km/h), as demonstrated when the estimated counts/30 s derived from the GAMM were plotted over temperature and windspeed (Figure 3). 

### 3.2. Pollen Pesticide Residues: Spring Pollination

**Figure 4:**
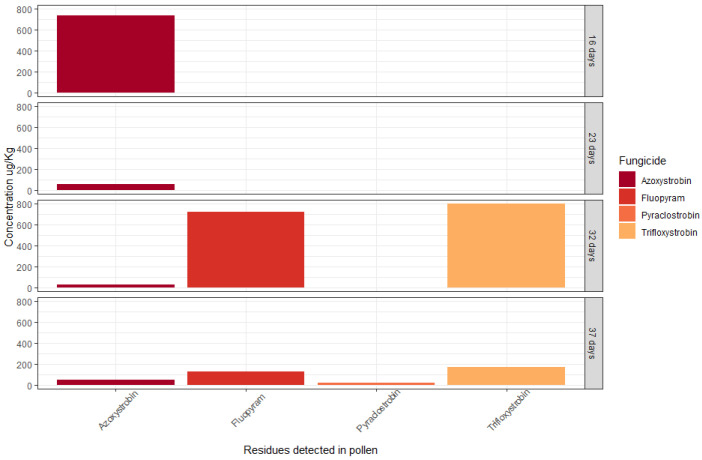
Concentrations (µg/Kg) of four fungicides detected in composite pollen sampled from hives situated in almond at 16, 23, 32, and 37 days after placement.

Four fungicides were detected in the comprehensive residue analyses of bee-collected pollen over 16, 23, 32, and 37 days after placement in the almond orchard. The fungicides included azoxystrobin, fluopyram, pyraclostrobin, and trifloxystrobin (Figure 4). The oral LD_50_ values for honey bees for these fungicides are: azoxystrobin (25 μg/bee), fluopyram (102 μg/bee), pyraclostrobin (110 μg/bee), and trifloxystrobin (110 μg/bee). The sum of pollen hazard quotient (PHQ) values, calculated based on these LD_50_ values, did not exceed the sublethal threshold of concern (PHQ = 50). The risk values for all detected residues by pollen sample were: PHQ = 29.6 (16 days), PHQ = 2.12 (23 days), PHQ = 14.88 (32 days), and PHQ = 4.94 (37 days).

### 3.3. Spring Pollination Activity

**Figure 5:**
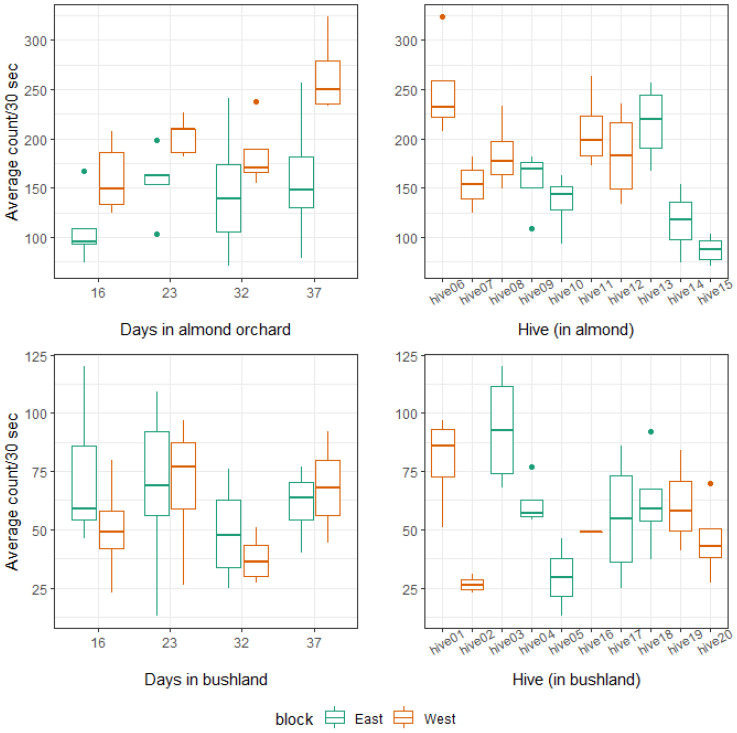
Mean honey bee counts per 30 s calculated in the period prior to the indicated days after placement, in either an almond orchard (top) or in bushland (bottom), and for average honey bee counts per 30 s for each hive in the study over all sampling intervals. Color legend indicates the direction that hives faced at the site (east or west).

Of the 20 hives placed in either almond or Mallee bushland, four collapsed—as indicated by lower average bee activity (counts/30 s) (Figure 5) and confirmed by visual inspection of the hives. These were hives 02, 05, and 16 in bushland and hive 15 in the orchard. Hives 02, 05, and 15 succumbed to undetermined diseases while hive 16 became a drone-laying colony and starved. The total average counts/30 s calculated for each hive, for days 16, 23, 32, and 37 after placement per site, showed activity differences between hives placed in almond compared to those in bushland, and activity was observed to increase over time for hives in almond only. 

The difference in mean bee counts/30 s between the sites was statistically significant (Table 2) when tested in the Poisson GLMM, with higher activity indicated for bee colonies placed in almond. No significant block effect was detected for hives facing east or west. Days after placement on site, as a fixed effect, significantly affected bee activity most notably between 16 and 23 days, as well as 16 and 37 days, respectively. The examination of residuals indicated a good fit of the data to the model. 

### 3.4. Summer Activity

**Figure 6:**
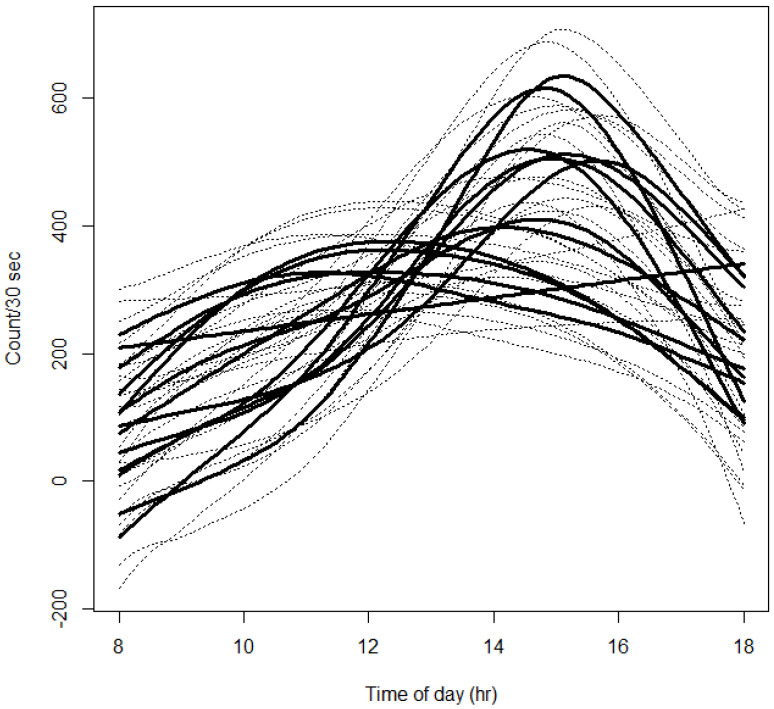
Hourly estimations of mean honey bee counts quantified for a subset of hives during summer to visualize daily activity, based on the Eyesonhives count. Plot generated from a fitted generalized additive mixed model (GAMM) and shows 95% confidence intervals for hive counts indicated with dashed lines.

Summer daily hive activity, when modelled using a GAMM, indicated that bee activity peaked between 1400 and 1600 h and was variable across hives at 1500 h (Figure 6). Subsequently, the time of 1500 h was selected for comparing differences in activity between hives previously placed in either almond or bushland. 

**Figure 7:**
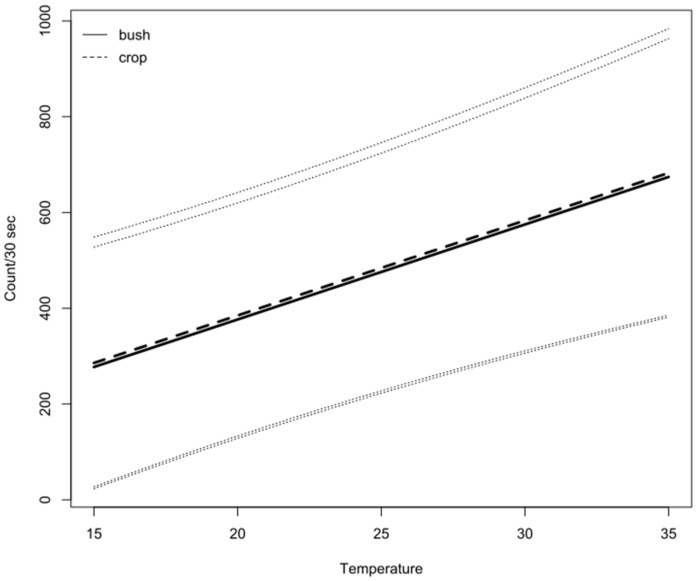
Estimated mean honey bee counts per 30 s with 95% confidence intervals, based on counts recorded at 1500 h, analyzed against temperature (°C) for hives that were monitored during the summer following pollination services of almond (crop) versus placement in Mallee bushland (bush).

During the summer monitoring, no significant difference in honey bee counts at 1500 h was found for hives that were previously located in almond (crop) or bushland (Figure 7). There was also no significant block effect for east- and west-facing hives in the summer (Table 3). 

### 3.5. Metabarcoding

There were 808,013 high-quality reads after processing, with an average length of 250 bp obtained for the ITS2 maker, experimental controls, and pollen sampled at four intervals from hives placed in either almond or bushland settings (Mallee/Sunraysia, Victoria) in the spring. Reads were divided among a total of 171 unique ASVs. 

Reads obtained from bee-collected pollen from hives situated in almond represented 42.1% of all reads in the study, of which 86.4% were assigned to *Prunis dulcis.* After the genus *Brassica* (exotic weeds), which contributed 9.4% of all reads from hives in the almond orchard, species from the genera *Eucalyptus* (Myrtaceae) contributed 1.9%, while *Acrtotheca* and *Sonchus* (Asteraceae; both exotic weeds) contributed 1.3% and 0.7% of reads, respectively.

Reads obtained from bee-collected pollen sampled in the bushland site represented 57.9% of all reads, excluding controls, of which *Brassica tournefortii* contributed 46.6% of the reads from bush samples, *Roepera* sp. (Zygophyllaceae) 28.7%, *Erodium* sp. (Geraniaceae) 7.1%, and *Medicago* and *Acacia* (Fabaceae) 6.0%. A further 4.5% of reads were assigned to the pollen substitute provided on site (*Glycine* sp.; *Saccharomyces cerevisiae*) and 0.5% of reads were assigned to *P. dulcis*, which were confirmed by comparing sequences to those obtained from the gDNA of an almond flower. However, the majority of *P. dulcis* reads detected were from pollen taken from hives at 16 days after placement in bushland, indicating likely background contamination from the pollen traps (the traps were used in previous seasons). After the initial sampling at 16 days, greater quantities of pollen were brought in by honey bees so that background contamination from the tray was not present or detected for 23, 32, and 37 day samples. 

For hives placed in bushland at Hattah, a total of 30 plant genera were identified from pollen DNA over 37 days, whereas a total of 16 genera were identified from pollen sampled from hives placed in almonds at Lake Powell for the same period. Following taxonomic assignment, the most abundant ASVs in the whole dataset were from almond *P. dulcis* with 38.7% of total reads, followed by *Brassica tournefortii* with 29.8% of total reads (family Brassicaceae otherwise represented 30.1% of all reads) and *Roepera* sp. (Zygophyllaceae; native) with 16.0% of reads. The percentages of ASVs by taxa appeared to correspond with the prevalence of flowering plants observed at the almond and bushland sites; however, pollen was not sorted by morphology in this study to enable a correlation analysis.

### 3.6. Alpha Diversity

**Figure 8:**
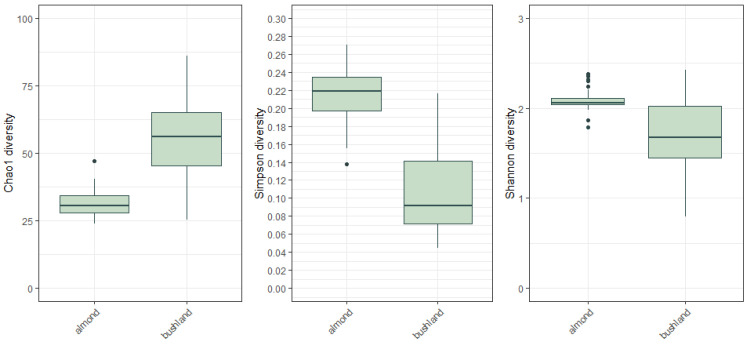
Alpha species diversity indices (Chao1, Simpson, Shannon) show differences for pollen taxa based on metabarcoding of pollen sampled for each site and over all days.

The alpha diversity measures indicated significant variability in pollen species richness between hives within sites, particularly for hives placed in bushland (Figure 8). Kruskal–Wallis rank sum test results of the Chao1 index was Χ^2^ = 43.52, df = 1, *p* < 0.01; for the Simpson index Χ^2^ = 21.69, df = 1, *p* < 0.01; and for the Shannon index Χ^2^ = 16.74, df = 1, *p* < 0.01.

### 3.7. Beta Diversity

**Figure 9:**
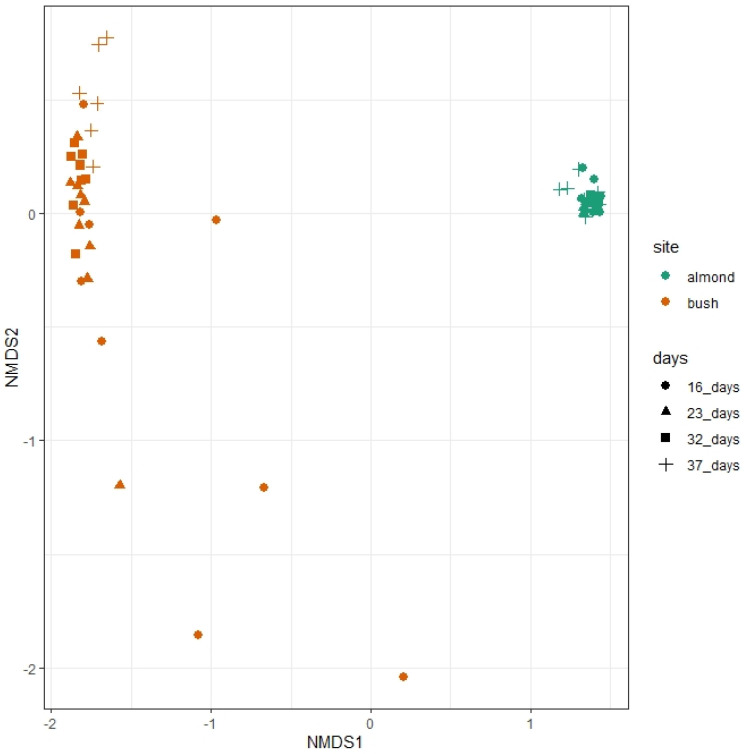
Non-metric multiple dimension scaling (nMDS) ordination plot showing dissimilarities in pollen species communities for hives placed in an almond orchard versus in a bushland. Communities are also grouped by sampling interval, which were 16, 23, 32, and 37 days after placement.

The Bray–Curtis two dimensional nMDS ordination demonstrate dissimilarities between pollen species’ community profiles based on the site of hives and the days after placement (stress = 0.063) (Figure 9). Across all data, PERMANOVA (adonis) showed significant differences between species profiles for pollen from the different sites (R^2^ = 0.673, df = 1, *p* < 0.01) and, over time, days after placement (R^2^ = 0.059, df = 3, *p* < 0.01), in addition to a significant interaction between the site and days (R^2^ = 0.059, df = 3, *p* = 0.03). However, the block effect representing hive position (cardinal direction) and number of bees (sum of flights per sampling interval) were not significant (R^2^ = 0.008, df = 1, *p* = 0.182), although there was perhaps a slight interaction effect on pollen species communities between the site and the number of bees (R^2^ = 0.01305, *p* = 0.079).

**Figure 10:**
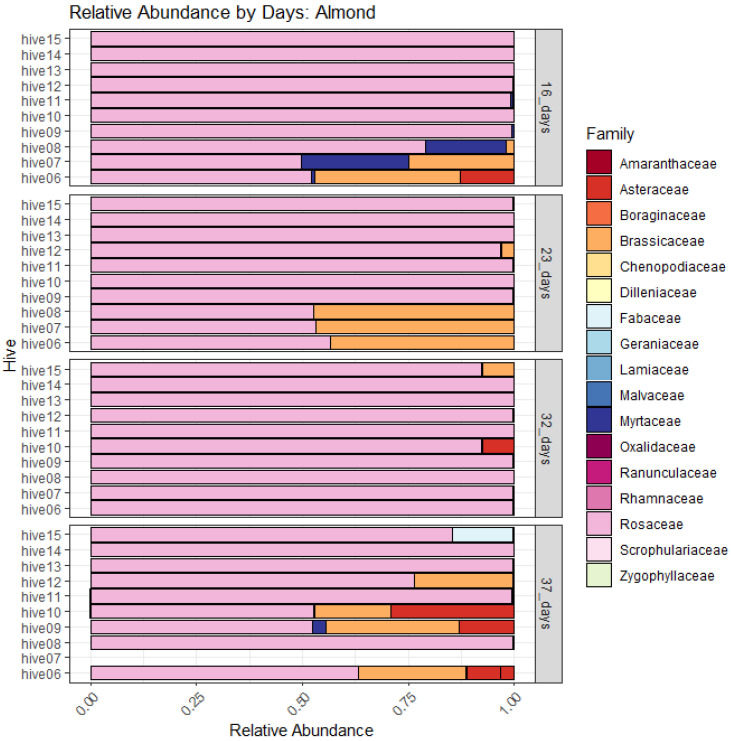
Composition of amplicon sequence variants (ASVs) concatenated by plant families from metabarcoding of the ITS2 marker based on honey-bee-collected pollen, sampled from hives at 16, 23, 32, and 37 days after placement in almond.

**Figure 11:**
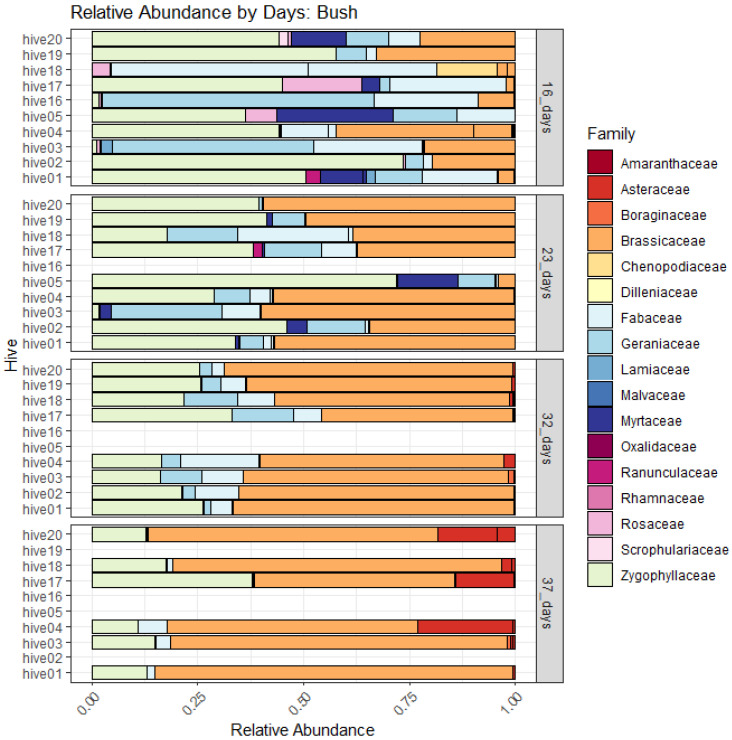
Composition of amplicon sequence variants (ASVs) concatenated by plant families from metabarcoding of the ITS2 marker based on honey-bee-collected pollen, sampled from hives at 16, 23, 32, and 37 days after placement in Mallee bushland.

When ASVs were merged by family to evaluate relative abundance between sites and among sampling intervals, the reliance of bees on pollen obtained from Myrtaceae at 16 days after placement in almond was evident, while pollen from Brassicaceae may have been important across all sampling intervals, except at ~32 days after placement (Figure 10). In contrast, the relative abundance of plant families was more diverse overall in pollen sampled from Mallee bushland, although bees were likely to be foraging predominantly on Brassicaceae species by 37 days (Figure 11). 

## 4. Discussion

Despite the reliance of the almond industry on managed honey bees for pollination in Australia, there has been surprisingly little field-based research published concerning the various factors known to adversely impact colony health and pollination efficiency [8,9,28,49]. We have shown that honey bee activity can be quantified in the field at the hive entrance to enable the evaluation of meaningful differential activity, using the Eyesonhives remote video monitoring system. The predicted number of honey bee flights was higher from hives that contained more frames of bees, based on the camera data, even after controlling for inherent variability in activity due to the time of day, local temperature, and windspeed. Furthermore, significant differential activity was observed over time between groups of hives of the same size placed in an almond orchard compared to the nearby Mallee bushland. Although there was approximately 60 Km distance between the sites, the local weather conditions recorded were similar. Differences in activity can, therefore, be reliably attributed to overall differences in the number of forager bee flights at each site. Initial validation using a tally counter indicated that the Eyesonhives counts (BPS) were overinflated compared to the manual counts; however, error in the regression model can be attributed to the difficulty in accurately counting by eye in ‘bees per second’, especially when there are more than six bees per second during a 30 s interval. Therefore, the tally counts were less reliable when a higher volume of bees where exiting/entering the hive compared to the camera data. 

Although our study is the first to demonstrate the use of the commercially available Eyesonhives technology specifically for research purposes, several studies have also shown that forager activity at the hive entrance can be monitored to evaluate hive status [50,51,52,53,54]. However, no other study has combined the use of video surveillance for activity with climate data, pesticide residue analyses, and DNA metabarcoding on bee-collected pollen as a suite of techniques to investigate multiple factors that can simultaneously affect colony health. Severe impacts on activity, such as colony collapse, were reliably detected using activity monitors by observing video footage but also when modelled remotely using processed count data. Bee activity data also showed healthy colony growth over time, which was validated by standard hive auditing (visual inspection of the number of frames of bees, data not presented). Although the Eyesonhives technology is likely impractical and expensive for use in large-scale pollination or honey production, the findings of this study indicate that there may be value in monitoring even a few colonies between different sites, as sentinel colonies, for the remote and rapid detection of important events, such as colony collapse resulting from communicable diseases or acute pesticide exposure. Recently, other inexpensive monitoring devices and protocols have been developed and reported to detect activity for exactly this purpose [55,56,57]. 

Four fungicide residues were detected in bee-collected pollen in varying concentrations between 27 July and 2 September 2019 or over 37 days placement of the study hives in the almond orchard. Although the concentrations of these residues were found to be low in terms of the risk of oral toxicity to honey bees, the possibility of synergistic effects of the specific fungicide cocktails were not assessed and may be important to investigate further [58]. Although fungicides are not usually considered to be acutely toxic to bees [59], active ingredients with low toxicity may pose a chronic risk to bee health if they are continually present in the foraging environment [60]. Furthermore, chronic fungicide exposure may increase the susceptibility of honey bees to pathogens [61,62]. Apart from the colonies that collapsed during our study, summer activity data indicated that surviving hives were at least able to recover from any chronic fungicide stress experienced because by December activity was comparable between all hives. During pollination, however, study constraints (i.e., the placement of hives and composite sampling) meant that honey bee activity could not be statistically compared (correlated) to pesticide exposure levels directly during the event. The raw data, when graphed, showed a potential block effect between west- and east-facing hives. Forager activity levels were higher on average for west-facing hives in the almond orchard, although this effect was not statistically significant and cannot be attributed to pesticide exposure anyway. It may be the result of afternoon sunlight casting shadows on hives during the time that juvenile bees were emerging for their orientation flights, although this effect was not observed in the bushland site for west-facing hives. It is important to note that the specific residues detected in pollen samples, azoxystrobin, fluopyram, pyraclostrobin, and trifloxystrobin may also reflect separate fungicide application regimes on different orchards because hives were situated at the border of two different farms, but pollen was pooled for analysis. The quantification of the possible synergistic effects of such fungicides on bees will require controlled experimentation under laboratory conditions. 

Pollen species diversity was perhaps less important for influencing forager activity and colony growth compared to pollen availability, as evident by the increase in activity observed in the almond orchard, specifically compared to bushland where the diversity of floral resources gathered by bees was found to be higher. However, in the bushland setting, floral resources may have been scarce in early spring, and, consequently, honey bees probably had to forage more widely when compared to those situated in the almond orchard. The extended flight duration of foragers to search for and collect pollen would likely be interpreted as lower activity levels at the hive entrance, when compared to bees that have access to an abundance of floral resources within a short distance to their colony. Future research to assess activity and pollen species diversity across multiple almond orchard sites, perhaps featuring different cultivars of almond trees, would enable a better understanding of the importance of pollen abundance compared to quality and/or floral diversity to enhance pollination efficiency. 

Frost, Collins, and Somerville [49] demonstrated that almond bloom progression is strongly correlated with almond pollen collection by honey bees in Australia. Although we did not quantify the amount of pollen collected in traps, based on pollen DNA metabarcoding results in our experiment, it may be inferred that the critical period of pollen collection for pollination occurred from 23–32 days after placement in the orchard (from approximately the 20th–30th of August 2019). However, the experiment would need to be repeated in subsequent seasons to ascertain an average critical period for pollinations services. The relative abundance of pollen taxa by the family in the 16- and 37-day samples was more diverse than the 23–32 day samples, indicating that bees foraged more widely at the onset of bloom and after 32 days in the orchard. For pollination services of almond, beehives are typically placed in the orchard just prior to bloom when floral resources are still scarce. The interval without much to forage on is typically brief; however, many hives arrive in the orchard already stressed from long-haul transportation in winter [8]. Therefore, flowering weeds (predominantly Brassicaceae) in orchard inter-rows and flowering *Eucalyptus* in hedgerows or other flowering remnant vegetation are important nutritional resources for supporting honey bees deployed for pollination services.

## 5. Conclusions

A key priority for achieving sustainable food production worldwide is to strike a balance between protecting crops efficiently against pests and diseases while maintaining healthy pollinator populations [63]. Although managed honey bees were the focus of this study, there are various reports suggesting that the factors that may impact honey bee colonies in agroecosystems, such as parasite and disease transmission, pesticide exposure, and climate extremes, may also ‘spill over’ or impact other invertebrate pollinators [48,64,65,66]. Although our study is preliminary, we have demonstrated the potential of using multiple approaches for a holistic evaluation of honey bee colony health in real time, when bees are active for pollination. Ultimately, the pollination efficiency of crops dependent on managed bees relies on retaining healthy colonies year after year; therefore, field-based research, such as this work, is important for the field of horticulture and for apiarists as it informs how to promote honey bee activity and health. 

## Figures and Tables

**Table 1:** Estimated regression parameters, standard errors, *t*-, and *p*-values for the Gaussian generalized additive mixed model conducted to compare bee activity for hive strength (strong/weak) based on colony size and to compare a sugar syrup supplement treated with chlorine versus no chlorine as a factor. Smoothing terms included climate variables for temperature and windspeed for each level of the hive strength factor.

	Estimate	Std. Error	*t*-Value	*p*-Value
Intercept	151.04	19.75	7.649	<0.01
Sugar supplement (treated)	30.29	21.85	1.386	0.166
Hive strength (weak)	−65.66	21.62	−3.036	<0.01
Smoothers	Factor	Effective df	F	*p*-value
Temperature (°C)	Strong	1	46.23	<0.01
Temperature (°C)	Weak	1	21.82	<0.01
Windspeed (Kmph)	Strong	5.65	24.18	<0.01
Windspeed (Kmph)	Weak	4.28	10.95	<0.01

**Table 2:** Estimated regression parameters, standard errors, z-values, and *p*-values for the Poisson generalized linear mixed model conducted to compare bee activity by site and days after placement. The estimated variance for the random effect ‘hive’ is 0.098.

	Estimate	Std. Error	Z-Value	*p*-Value
Intercept	4.872	0.125	39.059	<0.01
Site (bush vs. almond)	−1.088	0.143	−7.591	<0.01
Block (west vs. east)	0.136	0.143	0.947	0.344
Days (16–23)	0.236	0.031	7.729	<0.01
Days (16–32)	0.039	0.033	1.179	0.239
Days (16–37)	0.296	0.032	9.371	<0.01

**Table 3:** Estimated regression parameters, standard errors, *t*-values, and *p*-values for the Gaussian generalized additive mixed model conducted to compare bee activity for hives monitored during summer (post-pollination) for the cohort of hives placed previously in almond orchard versus in bushland settings. Smoothing terms included climate variables temperature and windspeed for the site factor.

	Estimate	Std. Error	*t*-Value	*p*-Value
Intercept	402.88	83.13	4.85	<0.01
Site (almond)	−3.56	105.23	−0.03	0.973
Block (west)	137.76	105.21	1.31	0.192
Smoothers	Factor	Effective df	F	*p*-value
Temperature (°C)	Bush	4.24	12.35	<0.01
Temperature (°C)	Almond	5.04	14.06	<0.01
Windspeed (Kmph)	Bush	6.27	4.92	<0.01
Windspeed (Kmph)	Almond	5.51	1.15	0.334

## Data Availability

The data presented in this study are available on request from the corresponding author. The data are not publicly available due to privacy.
